# Management and Outcomes of Descending Necrotizing Mediastinitis: A 15-Year Experience

**DOI:** 10.3390/jcm14051593

**Published:** 2025-02-26

**Authors:** Chirag P. Parjiea, Matti Sievert, Mohamed Anwar Haj Khalaf, Harald Ihmsen, Mostafa Higaze, Mika Gehrking, Andreas Wehrfritz, Horia Sirbu

**Affiliations:** 1 Department of Thoracic Surgery, Friedrich-Alexander-University Erlangen-Nürnberg (FAU), 91054 Erlangen, Germany; 2 Department of Otorhinolaryngology-Head and Neck Surgery, Friedrich-Alexander-University Erlangen-Nürnberg (FAU), 91054 Erlangen, Germany; 3 Department of Anaesthesiology, Friedrich-Alexander-University Erlangen-Nürnberg (FAU), 91054 Erlangen, Germany

**Keywords:** descending necrotising mediastinitis, thoracotomy, mediastinal debridement, tracheostomy, cervical debridement

## Abstract

**Background/Objectives**: Descending necrotising mediastinitis (DNM) is a severe, life-threatening infection that originates from the oropharyngeal or odontogenic regions and spreads to the mediastinum. It poses significant challenges due to its rapid progression and high morbidity. **Methods**: This monocentric, retrospective study analysed the records of 22 patients treated for DNM between 2008 and 2022. Diagnosis relied on characteristic clinical, radiological, and intraoperative findings linking oropharyngeal or cervical infections to mediastinitis. Contrast-enhanced computed tomography (CT) was used in all cases for diagnosis. Data collected included demographics, comorbidities, surgical interventions, time from diagnosis to surgery, re-operations, and complications. Microbiological analyses targeted aerobic and anaerobic pathogens. **Results**: The study included 22 patients (mean age 60 ± 9 years, 59% male) with DNM. The primary sources of infection were oropharyngeal (77%) and odontogenic (23%). Hypertension (86%), diabetes (68%), and cardiac arrhythmias (59%) were common comorbidities. Thoracotomy with mediastinal drainage and debridement was performed in 95% of patients, while 45% underwent cervicotomy and 82% required tracheostomy. The median intensive care unit (ICU) and hospital stays were 21 and 30 days, respectively. Delayed surgery (>24 h) significantly prolonged hospital stays (median: 62 vs. 28 days, *p* = 0.05). Re-operations were required in 82% of patients, with longer ICU stays observed in this group (median: 25 vs. 7 days, *p* = 0.003). Sepsis occurred in 55% and was associated with a higher tracheostomy rate (100% vs. 60%, *p* = 0.029). The mortality rate was 9%. **Conclusions**: Early recognition and prompt aggressive surgical intervention are paramount in managing DNM to mitigate complications and improve survival.

## 1. Introduction

Acute mediastinitis, a rare but fatal pathology associated with high morbidity and mortality, is defined as an inflammatory process of the mediastinum. Etiologically, mediastinitis can be classified as post-sternotomy-associated mediastinitis or deep-sternal wound infection (DSWI) in 0.2–5% of cases [1] and approximately 17% in cases of oesophageal perforation owing to traumata or iatrogenic causes [2,3]. In contrast, descending necrotising mediastinitis (DNM) represents a severe progression of infection originating from the head and neck regions, typically from an oropharyngeal or odontogenic source that spreads into the mediastinum. According to Endo’s classification, DNM can be divided into localised (Type I), diffused and extending to the lower anterior mediastinum (Type II) and diffused with involvement of the anterior and posterior lower mediastinum (Type IIb) [4,5].

DNM is caused by infections originating in the oropharynx (33–45%), odontogenic sources (26–47%), and the cervical region (15%) [4,5]. Historically, the mortality rate for DNM was up to 40% [6,7]. However, with advancements in diagnostics, the widespread use of antibiotics, improvements in radiographic imaging, and prompt surgical treatment, the mortality rate has decreased to 17.5% [8,9].

Due to the mediastinum’s proximity to the retropharyngeal space, infections can spread into the posterior mediastinum via the danger space, which extends from the sixth cervical to the first thoracic vertebra. Additionally, odontogenic infections may propagate to the anterior mediastinum through the lateral pharyngeal space, which connects to the retropharyngeal space. Furthermore, the anatomical continuity between the posterior pharyngeal, parapharyngeal, and submandibular spaces and the mediastinum facilitates the downward spread of infection. Once the infection reaches these spaces, its descent is driven by gravity, respiratory dynamics, and negative intrathoracic pressure, compounded by the absence of protective barriers within the fascial planes [10], further progressing the course of the synergistic effect that leads to the symbiosis of anaerobic and aerobic bacteria [11].

The gold-standard imaging modality for diagnosing DNM is computed tomography (CT) of the neck and thorax [12,13]. CT scans are crucial not only for the initial diagnosis but also for identifying residual abscesses and for ongoing surveillance throughout the treatment process [14]. While magnetic resonance imaging (MRI) provides superior soft-tissue contrast and can be useful in detailing the spread of the infection, its use is often limited due to longer acquisition time, making it less suitable for rapid diagnosis.

Therapeutically, DNM requires a multidisciplinary and comprehensive approach that includes initially empiric broad-spectrum antibiotics followed by adjustment of the antimicrobial agent based on the bacterial culture results. Prompt and aggressive surgical intervention remains a cornerstone, aiming to debride infectious tissue and provide infection drainage. Surgical strategies vary from minimally invasive techniques such as video-assisted thoracoscopic surgery (VATS) [15] to more extensive procedures such as bilateral cervical opening through cervicotomy, and thoracotomy [16,17]. The choice of surgical technique is influenced by the localisation of DNM and the presence of complications.

The study aims to investigate the patients treated at the Tertiary Division of Thoracic Surgery at the University Hospital Erlangen between 2008 and 2022 and identify factors that influence the outcomes and affect the therapy of DNM.

## 2. Materials and Methods

### 2.1. Study Design

We retrospectively analysed the medical records of adult patients (≥18 years) diagnosed and treated with DNM at our tertiary centre between 2008 and 2022. Patients were identified through a systematic review of electronic medical records through our in-house Electronic Health Record (EHR) system. Initially, we screened for a diagnosis of acute mediastinitis and further refined the selection to include only those meeting Esteras [6] diagnostic criteria for DNM. These criteria included (1) clinical presentation consistent with severe infection, (2) radiographic evidence of neck and mediastinal involvement, (3) intraoperative or microbiological confirmation of necrotising infection, and (4) a clearly established oropharyngeal or cervical source.

Patients with acute mediastinitis secondary to oesophageal perforation, post-sternotomy infection, or sternoclavicular joint osteomyelitis were excluded. The time of diagnosis was defined as the moment of presentation to our centre with concurrent positive clinical and radiological findings suggestive of DNM. Contrast-enhanced computed tomography (CT) was the imaging modality of choice for diagnostic confirmation and was performed in all cases.

Given the rare and life-threatening nature of DNM, this study did not include a comparative control group. A prospective study design with a control arm would have posed significant ethical concerns, as withholding or delaying aggressive surgical intervention is not justifiable in a high-mortality condition such as DNM. The heterogeneous clinical presentation and disease course among affected individuals also make establishing a well-matched control group difficult. Instead, our findings provide a descriptive analysis of real-world management strategies, with comparisons drawn from existing literature to contextualise outcomes.

### 2.2. Patient Characteristics

We collected data on patient demographics, including age, gender, and Body Mass Index (BMI), along with relevant comorbidities such as hypertension, diabetes mellitus, cardiac arrhythmias, and corticosteroid use. The clinical parameters recorded included the types of surgical interventions performed, such as thoracotomy, mediastinal debridement and drainage, and cervical debridement through cervicotomy and drainage. Furthermore, we documented the duration from diagnosis to surgery, perioperative complications like pleural effusion, pneumonia, empyema, and sepsis, and the length of stay in both the hospital and intensive care unit (ICU).

Risk stratification tools, including the Simplified Acute Physiology Score II (SAPS II) and the Sequential Organ Failure Assessment (SOFA) score, were incorporated into the analysis to assess disease severity and clinical outcomes.

The SAPS II is a validated scoring system that encompasses 17 variables, including physiological data such as heart rate, systolic blood pressure, body temperature, Glasgow Coma Scale, patient age, chronic health status and the type of ICU admission [18]. It is scored from 0 to 163 and predicts the likelihood of disease severity and mortality.

The SOFA score assesses the extent of a patient’s organ function or rate of failure over time, determining the morbidity rates in the ICU. The score is based on six parameters, namely, neurological, cardiovascular, respiratory, hepatic coagulation and renal systems. Each organ system is scored from 0 to 4, with an increasing score reflecting greater organ dysfunction [19].

### 2.3. Ethical Aspects

The study was conducted in accordance with the Declaration of Helsinki and approved by the Ethics Committee of Friedrich-Alexander University Erlangen-Nürnberg (Protocol Code 24-414-Br, 12 November 2024). Given the retrospective nature of this study, individual patient consent was waived as all data were collected as part of routine clinical care and anonymised for analysis.

All patient data were handled confidentially in compliance with institutional regulations and guidelines. No interventions beyond standard clinical care were performed, and no identifiable patient information is included in this publication.

### 2.4. Study Outcomes

The primary outcomes of this study were the duration of ICU and hospital stays. The secondary outcomes included surgical timing, defined as the duration from diagnosis to surgery (categorised as ≤24 h versus >24 h), the necessity for re-operations, the development of sepsis, the necessity for tracheostomy, and the mortality rate.

### 2.5. Surgical Approach

During surgery, the mediastinal pleura was opened in the upper and posterior mediastinum via anterolateral thoracotomy. A digital connection was created up to the cervical region. Thereafter, it was followed by aggressive mediastinal debridement and toilette. A 20-Charrière (Ch) Robinson drainage was placed, extending from the lower mediastinum to the upper mediastinum in the cervical region, positioned paratracheally, along with an additional Robinson drainage placed anteriorly.

**Figure 1:**
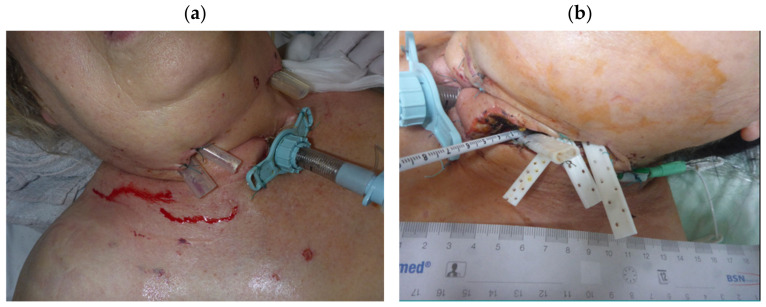
(**a**,**b**) Placement of cervical drainages.

Cervical drainage was performed via a bilateral transcervical approach. After sterile preparation, an incision was made along the anterior border of the sternocleidomastoid muscle on both sides, followed by platysma transection. The internal jugular vein, common carotid artery, and vagus nerve were identified and preserved. The retropharyngeal space was entered bilaterally, and blunt dissection was extended cranially and mediastinally to establish adequate drainage. Extensive irrigation and tissue debridement were performed, particularly in the deep mediastinal spaces and retropharyngeal space, and flat perforated drains were placed to ensure continuous drainage (Figure 1). The necrotic fascia was debrided, and the surgical site was thoroughly irrigated before closure.

### 2.6. Microbiological Workup

The microbiological evaluation was performed perioperatively to identify aerobic, anaerobic, and fungal pathogens involved in DNM. Intraoperative specimens were obtained from mediastinal abscesses, infected tissues, pleural effusions, and, when applicable, the retropharyngeal and cervical spaces. Additionally, bronchoalveolar lavage (BAL) fluid and blood cultures were collected in all patients with suspected systemic infection.

Samples were processed following standard microbiological protocols. Aerobic and anaerobic cultures were incubated using appropriate media, and bacterial identification was performed through biochemical assays and conventional microbiological techniques. Antibiotic susceptibility testing was conducted for all patients. In cases where fungal infection was suspected, yeast identification was performed using standard microbiological methods.

Empiric broad-spectrum antibiotic therapy was initiated upon suspicion of DNM, typically with Piperacillin-Tazobactam (4.5 g IV every 8 h) or Ampicillin-Sulbactam (3 g IV every 8 h). Antimicrobial therapy was later adjusted based on culture and susceptibility results. Patients with positive blood cultures or polymicrobial infections were managed in consultation with the in-house microbiology department to optimise antimicrobial therapy.

### 2.7. Follow up

Patients were monitored throughout their hospital stay, with follow-up continuing post-discharge. When oral intake was not feasible, nutritional support was provided via nasogastric tube or percutaneous endoscopic gastrostomy (PEG). Haemodynamic management and intensive care followed sepsis management principles, with treatment strategies evolving in accordance with contemporary guidelines during the study period [20,21]. Pain control was achieved using opioids such as piritramide, tramadol, and buprenorphine, as well as non-opioid analgesics, including paracetamol, metamizole, and ibuprofen. In cases of chronic pain, the Department of Pain Management provided additional co-management.

Patients were discharged once they were clinically stable, defined as being afebrile for at least 72 h, haemodynamically stable without vasopressors, and demonstrating adequate respiratory function without invasive mechanical ventilation. A resolution of infection was confirmed by decreasing inflammatory markers, including C-reactive protein (CRP) and white blood cell (WBC) count, alongside the absence of new infectious collections on imaging. Additionally, wound healing had to be satisfactory, with no evidence of active drainage or infection. After discharge, patients were typically transferred to pulmonary rehabilitation facilities or weaning clinics for further recovery and supportive care.

Patients were scheduled for a routine ambulatory follow-up visit 10 days after discharge to evaluate their clinical recovery, surgical wound healing, and inflammatory markers. Depending on individual recovery trajectories, further outpatient visits were arranged as necessary. Imaging, including chest X-rays or CT scans, was conducted selectively in cases where residual infection or recurrence was suspected.

### 2.8. Statistical Analysis

Data analysis was conducted using Statistical Package for the Social Sciences (SPSS, version 28.0, IBM Corp., Armonk, NY, USA). Descriptive statistics were used to summarise all continuous and categorical variables, with continuous variables presented as mean ± standard deviation (SD) or median and range and categorical variables presented as frequencies and percentages. For comparisons between groups, continuous data were assessed for normality using the Shapiro–Wilk test, and depending on the normality of the data, comparisons were made using either a *t*-test for normally distributed variables or a Mann–Whitney U-test for non-normally distributed variables. Categorical data were compared using Fisher’s exact test to assess differences in proportions between groups. Changes in parameters over time, such as the change in SOFA scores from the first to the last day in the ICU were tested for statistical significance using the Wilcoxon signed-rank test for paired samples. A *p*-value of <0.05 was considered statistically significant.

## 3. Results

### 3.1. Demographics and Risk Factors

Twenty-two patients diagnosed with DNM at the Tertiary Division of Thoracic Surgery, University Hospital Erlangen, between January 2008 and December 2022 were included in this study. Patients’ demographics and comorbidities are summarised in Table 1. The most prevalent comorbidities included hypertension, diabetes mellitus, and cardiac arrhythmia.

**Figure 2:**
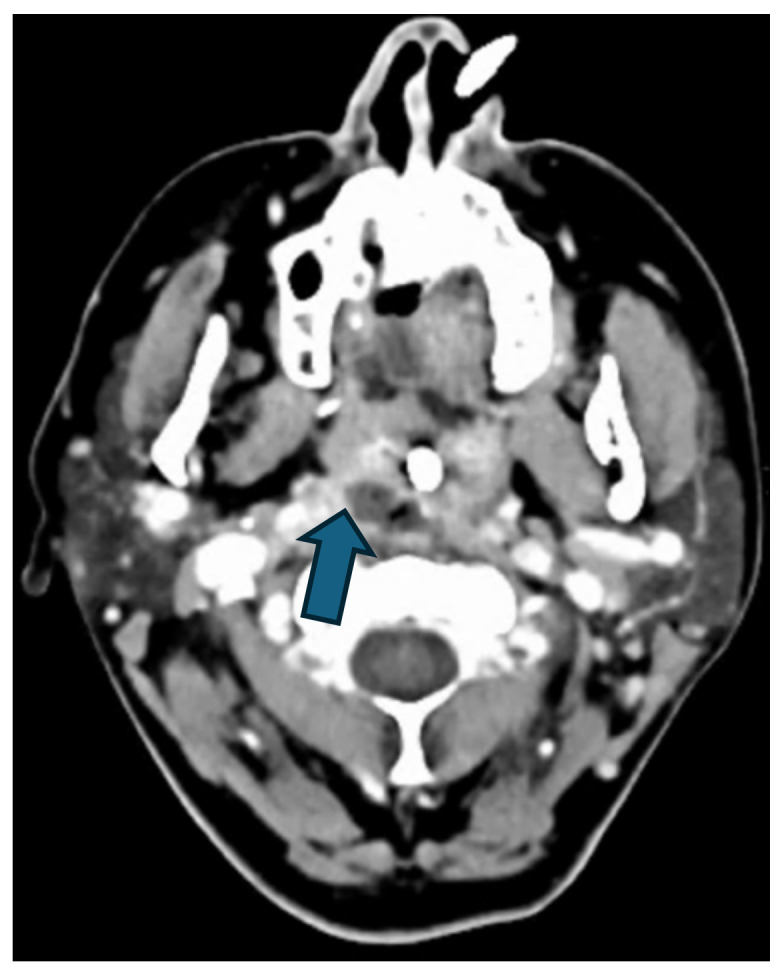
Hypodense formation (blue arrow) with peripheral contrast enhancement in the right retropharyngeal region, beginning paramedian caudal to the right pharyngeal tonsil/at the level of the C2 vertebral body, with gas inclusion in a patient with retrotonsillar abscess.

**Figure 3:**
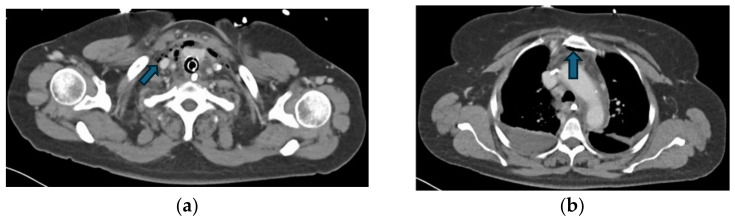
CT findings of the same patient presented in Figure 2, demonstrating the progression of DNM. (**a**) Gas inclusions (blue arrow) extending along the platysma muscle and thyroid gland into the anterior mediastinum; (**b**) Gas inclusions (blue arrow) posterior to the manubrium sterni with right sided pleural effusion.

Regarding the source of infection, 77% (n = 17) of patients had DNM originating from oropharyngeal infections, while 23% (n = 5) had odontogenic sources. All patients underwent CT scans for the primary diagnosis (Figure 2 and Figure 3), and if required, serial CTs were performed to monitor the disease’s progression.

### 3.2. Surgical Interventions, ICU, and Hospital Stay Course

Surgical interventions are presented in Table 2. The primary surgical approach for most patients was thoracotomy, with mediastinal drainage and aggressive debridement. Cervicotomy was performed in 45% (n = 10) of patients with the aim of cervical debridement, and 82% (n = 18) required a tracheostomy during their hospital stay.

The median length of ICU stay was 21 (2–95) days, and the median length of total hospital stay was 30 (14–123) days. 82% of the patients (n = 18) required re-operations, with the number of re-operations ranging from 0 to 6 (median: 2). Re-operations were often necessary to control ongoing infection and manage complications such as pleura empyema or recurrent infections. For patients requiring re-operations, the length of ICU stay was significantly longer, while the length of total hospital stay did not differ (Table 3).

Patients who had surgery more than 24 h after diagnosis required longer hospital stays compared to those who underwent surgery within 24 h, while the length of ICU stay did not differ (Table 4).

### 3.3. Perioperative Complications

Perioperative complications were common in this cohort. Pleural effusion, pleural empyema, sepsis, and pneumonia were the most frequent complications (Table 5).

There was a significant association between sepsis and the need for an early tracheostomy, while the lengths of ICU stay and hospital stay were not affected by sepsis (Table 6).

### 3.4. Microbiological Findings and Antibiotic Treatment

Microbiological analysis revealed a polymicrobial infection pattern, with both anaerobic and aerobic bacteria commonly isolated (Table 7). These findings emphasise the diverse microbiological nature of DNM and the need for broad-spectrum antimicrobial therapy to effectively address both anaerobic and aerobic pathogens (Table 8). Blood cultures were positive in three patients (14%).

### 3.5. Risk Stratification Scores and Outcomes

The initial SOFA score was calculated for all patients on admission to the ICU. The mean SOFA score was 7 ± 4 (range: 2–16). The SAPS II score was 42 ± 17 (range: 20–88). During the ICU stay, the SOFA score significantly decreased, from a mean of 7 ± 4 on Day 1 to 3 ± 2 on the last recorded day (*p* < 0.001), indicating improved organ function with treatment. The overall mortality rate was 9.1% (n = 2).

## 4. Discussion

Our study analysed 22 patients over a 15-year period and found that oropharyngeal infections (77%) were the leading cause of DNM. Despite the high morbidity associated with the disease, our cohort demonstrated a lower mortality rate (9%) compared to historical data. Delayed surgical intervention (>24 h after diagnosis) significantly prolonged hospital stays (median: 62 vs. 28 days, *p* = 0.05) and increased ICU burden, reinforcing the importance of timely debridement and drainage. Sepsis occurred in 55% of cases, correlating with a higher tracheostomy rate (100% vs. 60%, *p* = 0.029). The need for re-operations was high (82%), underscoring the complexity of infection control and disease progression.

The mean age of patients in our study was 60 ± 9 years, similar to the findings of Chen et al. (57.8 ± 15.2 years) [22] and Ho et al. (56.7 ± 22.2) years [23]. Gender distribution in our cohort showed a male predominance (59%), which aligns with recent studies [12,13,16,22,24]. Regarding the aetiology of DNM, our findings demonstrated a prevalence of oropharyngeal infections, while odontogenic sources accounted for 23% of the cases. This differs from studies in other regions, where odontogenic infections are more prevalent. Recent data suggests that odontogenic infections were the primary source of DNM in 45.2% to 62.5% of the cases [12,25].

The keystones of DNM therapy are the rapid elimination of its cause, aggressive surgical drainage, and extensive debridement. Surgical options range from cervicotomy and VATS to thoracotomy, based on the localisation of the infection. Our study predominantly utilised anterolateral thoracotomy (95%) for mediastinal drainage and debridement, with cervicotomy (45%) performed as needed. This aggressive approach aligns with previous studies that emphasise the necessity of extensive surgical debridement in advanced DNM cases. Similar findings were reported by Congedo et al. [26], where thoracotomy combined with cervicotomy was required in 50% of cases, while VATS was used in 22.2%. Likewise, Leonardi et al. [15] found thoracotomy to be the predominant approach (71%), with only 29% of cases treated via VATS, noting a shorter ICU stay and surgery time in the VATS group.

While minimally invasive techniques such as VATS have been increasingly explored, their effectiveness remains controversial. Tanaka et al. [27] compared VATS and thoracotomy in DNM and found no significant differences in mortality but noted a higher postoperative complication rate and increased reoperation rates in the VATS group (37.9% vs. 15.5%). These findings suggest that while VATS may offer reduced surgical invasiveness, it may not provide adequate mediastinal access for extensive debridement and adhesiolysis, particularly in cases with deep-seated infections, as corroborated by other authors [28,29]. Alternatively, the study by Zhao et al. [12] highlighted the potential role of vacuum-sealing drainage (VSD), which was applied in 35.5% of the cases and associated with shortened ICU and hospital stays. Although our cohort predominantly underwent open surgery, these findings reinforce the role of minimally invasive and adjunctive drainage techniques in DNM management.

Localised infections (DNM Type I), according to Endo classification [5], were treated with cervicotomy for debridement, drainage and placement of cervical drains. This approach is generally effective for infections confined to the upper mediastinum [4,6,25,28,29]. A multicentre observational study by Sugio et al. [30] analysed 225 patients and demonstrated that in type I cases, cervical drainage was the most commonly used method (34.3%). In contrast, Misthos et al. [31] reported that less invasive surgical approaches, such as isolated cervical drainage, were associated with high reoperation rates and unsatisfactory outcomes.

Tracheostomy remains a controversial topic due to concerns about downward infection spread into the mediastinum [7,8]. Kim et al. [32], reported that tracheostomy is a safe and effective method for airway management. Similarly, Chen et al. [33] described that tracheostomy in deep neck infections facilitates respiratory care without increasing the risk of infection spread. In our cohort, none of the patients who underwent surgical tracheostomy experienced contamination or infection at the tracheostomy site, aligning with findings from Chen et al. and Kim et al.

The management of DNM requires a multidisciplinary approach, integrating airway security, infectious disease management, and intensive care support. Ho et al. [23] observed the benefits of early coordinated intervention involving otorhinolaryngologists, thoracic surgeons, and infectious disease specialists, reporting a mortality rate of 9.5%. Their findings also highlight the role of tracheostomy (28.5%) in airway management and the need for repeat surgical interventions (61.9%), reinforcing the necessity of staged debridement. These results underscore the role of multidisciplinary, protocol-driven care in optimising DNM management.

The timing of surgical intervention in patients with DNM significantly impacts both hospital and ICU outcomes and patient prognosis. Jablonski et al. [34] found that early surgical intervention (<24 h after diagnosis) was associated with lower mortality, reinforcing the importance of timely debridement and drainage to improve mortality. Their study also demonstrated that delayed surgery imposed a mortality rate of 31.8% across different aetiologies of acute mediastinitis.

Our microbiological analysis confirmed DNM as a polymicrobial infection dominated by *Streptococcus* species (32%) and *Staphylococcus* species (27%), consistent with the oral cavity’s microflora. However, recent advances in metagenomic next-generation sequencing (mNGS) have provided deeper insights into the microbial diversity in DNM. Sun et al. [35] demonstrated that mNGS could rapidly identify *Streptococcus anginosus*, *Prevotella oris*, and *Mogibacterium timidum* as dominant pathogens, while also correlating *S. anginosus* abundance with inflammatory markers such as CRP and procalcitonin. This technique enhances pathogen detection beyond conventional culture, especially for fastidious anaerobes. Furthermore, Brajkovic et al. [36] reported the presence of multidrug-resistant ESKAPE pathogens (*Enterococcus faecium*, *Staphylococcus aureus*, *Klebsiella pneumoniae*, *Acinetobacter baumannii*, *Pseudomonas aeruginosa*, and *Enterobacter* species), emphasising the increasing role of antibiotic-resistant bacteria in DNM. These findings reinforce the need for rapid molecular diagnostics, such as mNGS, which has been shown to improve pathogen detection and antimicrobial stewardship in DNM. Given the rise in resistant organisms, empirical antibiotic regimens should include coverage for anaerobes and multidrug-resistant aerobic pathogens, with early de-escalation based on culture results.

The mean SOFA score at ICU admission (7 ± 4) indicated significant organ dysfunction, while the SAPS II score (42 ± 17) reflected moderate to severe illness. Importantly, we observed a significant decrease in SOFA scores over time (*p* < 0.001), correlating with clinical improvement. Palma et al. [37] found that higher SAPS II scores were significantly associated with increased mortality (*p* < 0.01), and a delay in ICU admission correlated with higher SAPS II scores and longer ICU stays. Similarly, Ramos-Hinojosa et al. [38] demonstrated that total SOFA scores, particularly the respiratory and cardiovascular components, were strong predictors of mortality in DNM patients, with an in-hospital mortality rate of 33.3%. Further research into these methods of risk stratification could help refine early prognostic assessment, optimise ICU resource allocation, and improve patient outcomes in DNM.

This study has several limitations that should be acknowledged. The small sample size, inherent to the rarity of DNM, limits the statistical power of the analysis and may explain the lack of statistical significance in some comparisons. Furthermore, the high inter-individual variability in clinical presentation, comorbidities, and management approaches add complexity to data interpretation and may reduce the generalizability of the findings. A comparative control group was not included due to ethical and practical constraints. Given the life-threatening nature of DNM, withholding or delaying aggressive surgical intervention for the sake of comparison would be unethical and unfeasible. The retrospective design also introduces potential biases, including relying on the accuracy and completeness of historical medical records.

Additionally, as a single-centre study, the results reflect the protocols and expertise available at our institution and may not apply universally to other healthcare settings. This was not a confirmatory but an exploratory study. Therefore, larger, multicentre prospective studies are required to prove the present findings with higher statistical evidence.

## 5. Conclusions

Early recognition and prompt aggressive surgical intervention are paramount in managing DNM to mitigate complications and improve survival. Delayed surgery beyond 24 h was associated with a significantly prolonged hospital stay. Thoracotomy remained the preferred approach for extensive mediastinal debridement, while cervicotomy was useful in localised infections. Our findings highlight the importance of a standardised surgical approach and early airway management. Further research with larger cohorts is warranted to refine treatment strategies and improve long-term prognoses, as no standard of care is yet fully established.

## Figures and Tables

**Table 1:** Patient Demographics (n = 22) and Comorbidities.

Characteristic	Value
Mean age (years)	60 ± 9
Male/Female	59%/41%
Hypertension	86% (n = 19)
Diabetes Mellitus	68% (n = 15)
Cardiac Arrythmias	59% (n = 13)
Corticosteroid use	18% (n = 4)

**Table 2:** Surgical Interventions.

Procedure	Percentage (%)
Thoracotomy	95% (n = 21)
Mediastinal Drainage	95% (n = 21)
Mediastinal Debridement	91% (n = 20)
Cervicotomy	45% (n = 10)
Tracheostomy	82% (n = 18)
Re-operations required	82% (n = 18)

**Table 3:** Length of ICU stay and hospital stay in patients requiring re-surgery.

Parameter	Re-Surgery	Median (Range)	*p*-Value
ICU stay (days)	Yes (n = 18)	25 (2–95)	0.003
	No (n = 4)	7 (4–10)	
Hospital stay (days)	Yes (n = 18)	31 (14–123)	0.39
	No (n = 4)	24 (18–48)	

**Table 4:** Length of ICU stay and hospital stay vs. timeliness of surgery.

Parameter	Diagnosis to Surgery	Median (Range)	*p*-Value
ICU stay (days)	≤24 h (n = 18)	19 (4–95)	0.34
	>24 h (n = 4)	42 (2–80)	
Hospital stay (days)	≤24 h (n = 18)	28 (14–94)	0.05
	>24 h (n = 4)	62 (30–123)	

**Table 5:** Perioperative Complications.

Complication	Incidence (%)
Pleural Effusion	64% (n = 14)
Pleural Empyema	64% (n = 14)
Sepsis	55% (n = 12)
Pneumonia	27% (n = 6)

**Table 6:** Sepsis and outcome.

Parameter	Sepsis	Value	*p*-Value
ICU stay (days)	Yes (n = 12)	27 (2–80)	0.28
	No (n = 10)	14 (4–95)	
Hospital stay (days)	Yes (n = 12)	29 (14–82)	0.67
	No (n = 10)	31 (18–123)	
Tracheostomy	Yes (n = 12)	100% (n = 12)	0.029
	No (n = 10)	60% (n = 6)	

**Table 7:** Most common microorganisms isolated.

Microorganism	Prevalence (%)
*Streptococcus* spp. (*oralis*, *constellatus*, *pneumoniae*, *angionius*)	32%
*Staphylococcus* spp. (*aureus*, *epidermidis*)	27%
*Pseudomonas aeruginosa*	18%
Others (*Eggerthella lenta*, *Actinomyces meyeri*, *Escherichia coli*, *Parvimonas*, *micra*)	23%
*Candida* spp.	9%

**Table 8:** The most common types of antibiotics used.

Antibiotic Class	Antibiotic	Prevalence (%)
Beta lactam	Piperacillin-tazobactam	90%
Carbapenem	Imipenem/Meropenem	40%
Lincosamides	Clindamycin	30%
Cephalosporins	Cefazolin	40%
Nitroimidazoles	Metronidazole	20%

## Data Availability

The data presented in this study are available at the reasonable request of the corresponding author.

## Abbreviations

The following abbreviations are used in this manuscript:

DNM	Descending Necrotizing Mediastinitis
BMI	Body Mass Index
CT	Computed Tomography
SOFA	Sequential Organ Failure Assessment
VSD	Vacuum-sealing drainage
SAPS II	Simplified Acute Physiology Score II
PEG	Percutaneous Endoscopic Gastrostomy
EHR	Electronic Health Record System
Spp	Species (plural)
CRP	C-Reactive Protein
WBC	White Blood Cells
Ch	Charrière (French gauge)
ICU	Intensive Care Unit
mNGS	Metagenomic next-generation sequencing
DSWI	Deep Sternal Wound Infection
